# Selections and Abstracts

**Published:** 1902-02

**Authors:** 


### SELECTIONS AND ABSTRACTS.

---

### ORGANIZATION.

The present time not only appears to be, but is, an actual era of organization, which, being in trend of thought, may be recognized and felt in the air that is breathed.

In this city a strike of labor against capital in one of the chief industries of the city has been going on for some weeks. It is sufficient to afford a practical illustration. Singly and alone neither party in interest could have hoped to attain the ends so ardently sought; with a complete organization of those who were individually interested in attaining the purposes sought there is hope.

The men, upon their side, have presented a very solid, united front to their former employers, while the manufacturers hold together in quite as complete an opposition. Both parties were and are thoroughly organized for ends specified. Which party will eventually succeed is difficult to prognosticate.

Every one knows of the great capitalistic trusts and combinations of corporations which have sprung into active existence within the past few months, which are wielding a power in society, state and nation that is as formidable as an army with banners.

The inherent strength of these great forces, that are so strong as to be almost irresistible, is found in the one expressive word, "confidence." Without this element every one of them would speedily segregate and go to pieces. Mutual interests and aims, diverse as they are, bind one element to the other with hooks and strands of toughest steel. These great organizations are around and about on every hand and in all directions, furnishing object-lessons for all who are inclined to learn.

The medical profession of America has been in process of organization for more than half a century, beginning with the great national body, the American Medical Association. To be sure, a very few of the States and more prominent cities had a semblance of local organization, but the time appeared a long way off before there would be a systematic organization that would be fairly representative of the medical profession in all of its component parts, such as may be seen and observed at this time.

The American Medical Association has grown numerically until it has become a great power as a professional body. The State societies are not correspondingly strong. To be sure, there are many efficient, useful, working State associations, but there are scarcely any that are anywhere near the high-water mark as influential bodies, and one cogent reason may be found in a usual lack in the formation of county societies. These are the fundamental bodies that need care and nurture. Even in the populous State of Ohio there are some counties without a society. The same may be stated as true of many counties in Kentucky and other States south of the Ohio river.

In this connection it is a real pleasure to state that in some sections there is quite a revival of interest in the county societies, but much remains to be done. One good executive officer to act as secretary, who has a lively interest in the welfare of his profession, will be able to inaugurate and keep a county medical association in good working order. The sending out of notices of next meeting is very important; that and the keeping of the minutes are the all-important functions of this officer. If there is a president who will go to a little trouble in soliciting papers and seeing to it that discussions are taken up and carried forward with vim, no society so constituted can fail.

The next thing is to get all the physicians in a county to come in for identification; some may object to a taking in of those who, from ignorance or other cause, have gone astray and have not been in the past all that is commendable as practitioners. Unless such persons have been guilty as criminals it is very much better to take them in, and, if necessary, reason with them as to their shortcomings, and through the beneficent influences of the society endeavor to make better men of them. In almost every county there are one or more to whom some minor exceptions may be taken—many times an outgrowth of envy or exaggerated statements pertaining to some one who is unhappily possessed of a supersensitive nature, for which he is not particularly to blame, having been born so. However, the pronounced tendency of the times is towards a liberal charity in these directions, which is well.

The recently enacted laws in many of the States make the recognized physicians of the Eclectic and Homeopathic schools legal practitioners, which, as it is seen to-day, is a good move, and in the right direction. Most of these men are good, true and honest, with a social and moral standing that is equal to the best in the land. They have had a creditable education, and if they do not see things as they appear under my microscope, that is no valid reason why I should ostracise myself or them even in a professional way, which leads to a thought that such men should be taken into so-called regular societies whenever they can be induced to apply for membership, and when enrolled respect their recognized professional training. The statement is often iterated that a man is either a knave or a fool to hold given doctrines. Not so. That is his birthright, and who is to say that he is not just as honest and has as much right to practice in accordance with those opinions as the one who indulges in captious criticism?

The period of pharisaism in medicine has almost disappeared, and it will soon be historically reckoned with the "has beens." So the suggestion is made to invite all such men as may be legal practitioners to become members of the county medical societies. It will do any county society a world of good to take this course. There is not a man who has ever served on what are called mixed State boards in any of the States who does not think just this way.

The band-wagon is within hearing distance, and this is a right good time to prepare to get in, so that the golden opportunity of enjoying a ride all together may be taken advantage of. Such action upon the part of the county medical societies will be sufficient to start a real true-blue revival, in which every one will wonder why this was not thought of before.

There are lots of things that are now expedient that were not so considered even a few years ago. Rapid transit, making it easy to get together, has had much to do with changing men's views of every conceivable subject. To get together is the greatest thing of all, and when together it is wonderful how individual and personal opinions are modified and often disappear when adjudged absolutely insurmountable.

The writer always enjoyed observing the comparative unselfish giving way to and respecting the rights of others in members of large families, and a corresponding sympathy with and for an only child. The medical profession is broad, deep, high and wide enough to take in and embrace every man who has been determined by the laws of the land to be a legal practitioner. The men referred to are not charlatans or quacks, but men who accepted certain theories in regard to practice. They had a good and inalienable right to do this, and now that our horizon of view is so cleared and widened, there appears in the offing an opportunity to do some readjusting that will be creditable to all concerned.—*Cin. Lancet Clinic.*

---

### FAILURE OF THE KNIFE IN THE TREATMENT OF CANCER.

For many years the writer advocated the thorough removal of all cancerous tumors by the knife. In the recent years of his professional career, however, the discouraging fact of the almost invariable return of these tumors, after being operated upon, has greatly modified his views as to the benefits to be derived from these operations.

The following opinions of authorities will show that other surgeons have had similar experiences: Dr. J. M. Baldy, of Philadelphia, in a paper read at the last meeting of the American Medical Association, says that only 5 per cent. of the patients operated upon for cancer of the cervix uteri are cured. He further states that the statistics of the operations at Johns Hopkins Hospital gave the same result. In an abstract of the views of P. G. Unna on the subject of cutaneous cancer, we find that he recommends the use of the Paquelin cautery as a substitute for the knife. This is generally used superficially, singeing the parts only. He states that he has observed a checking of the malignant process, and the appearance of granulation tissue. Herzfield says that abdominal hysterectomy for uterine cancer is a failure, for the cellular tissue and lymph glands cannot be removed. Vaginal hysterectomy, he says, is only palliative in 50 per cent. of the cases operated upon; it is, however, absolutely without danger, and in the remaining cases lasting results are obtained.

At the Ninth German Congress of Gynecology, held at Giessen, May 29-31, 1901, Professor W. A. Freund said that he was able to report only two cases of cancer of the uterus permanently cured by surgical operation in twenty-three years of experience. He learned by inquiry in the various clinics of Germany that the average mortality, after abdominal extirpation with removal of the larger portion of the parametrium and the glands, is 24.6 per cent., and with a record of 46.6 per cent. of recurrences during the first year.

Dr. Cyrus A. Kirkley, at the last meeting of the American Gynecological Society, May 30 to June 1, 1901, said that the only indication for abdominal hysterectomy was when the cancer was strictly confined to the cervix and body of the uterus, or to the body alone. Electro-cauterization, as practiced by Dr. Byrne, was given the preference over all other methods of operating.

In an editorial note occurs the following: "Carcinoma of the uterus and its treatment has been the subject of thorough discussion in the American and German gynecological societies during the past few weeks. All the reports in regard to the treatment and recurrence are most melancholy and unsatisfactory."

An article by Dr. C. Trunecek on the mechanism of the arsenical treatment of malignant tumors is commented on as follows: "He now announces that carcinoma or sarcoma cells are directly necrosed by the action of the arsenic, and their protoplasm is coagulated. The stroma cells become degenerated and exudation follows, which in turn produces further changes in the tumor cells. Inflammation with demarkation is the result of the application of arsenic to the surrounding sound tissue. It gradually passes into into suppurative stage, terminating in the throwing off of the entire necrosed tumor as a foreign body. In short, the organism reacts to the arsenical tumor as to a foreign body, and throws it off. The tumor when left alone does not induce any reaction of this nature; the organism makes no effort to defend itself against it, and is in turn destroyed by it."

The universal use by all surgeons of the principles of aseptic surgery has rendered the operations for cancer so comparatively safe that surgeons have been tempted to prevent the recurrence of these tumors by extending the field of their operations. So extensive are the mutilations now recommended by some authors for the removal of cancerous tumors, especially of the uterus and its appendages, that it would seem doubtful whether we ought not to describe the operation as one of the removal of the patient from the tumor rather than that as one of removal of the tumor from the patient.

What does this show? It seems to me that it is a virtual confession of the failure of the knife in the cure of these cases, in that we are extending more and more our incisions into the apparently healthy tissues, in order to prevent this recurrence. Admitting, therefore, for the sake of argument, that the use of the knife has been a failure in these cases, what is the reason for it?

After a prolonged study of this subject the followiug explanation has suggested itself to me, as seemingly conclusive on this point. When we make an incision into any part of the body for the removal of a malignant growth, we at once divide and lay wide open for infection every vein and lymphatic vessel in the part operated upon. No matter what we may believe to be the contagium of cancer, the fact remains the same. Dr. Lambert Lack, of England, believes that cancer is produced by an abnormal development taking place in normal or healthy epithelium when invading the lymph spaces of the body. In confirmation of this theory, he adduces the results of his experiments with rabbits and other animals. In a number of cases, by introducing healthy epithelium into the abdominal cavities of rabbits, he succeeded in developing cancerous tumors.

Dr. Konstantinowitch succeeded in producing growths not dissimilar from granuloma, and containing epithelioid and giant cells, by inserting spores of lycopodium under the skin. Dr. Harvey Gaylord and others assert, on the other hand, that cancer is produced by the growth in the body of a living organism—a protozoon.

Whatever theory we may adopt as to the causation of cancer, there are two facts in its history that seem to be now generally admitted. The first of these is that it is probably always local in its early stages, and the second is that its origin is due to an injury or local irritation of the parts affected. If we believe that the recurrence of cancer in cases operated upon is due to infection through the medium of the divided veins and lymph channels, the question arises, Can we supplement the knife, or substitute for it any other plan for removing these tumors that will prevent this systemic infection ?

It is the belief of the writer that this can be done by adding to, or substituting for the knife the electric cautery, the thermocautery, or, in a limited number of cases, the use of arsenic or chloride of zinc for the removal of the morbid growths. The marvelously successful results of Dr. John Byrne, of Brooklyn, in the treatment of uterine cancer show clearly that the removal of uterine cancer by the electric cautery is, by far, the best method of treatment.—*Robert A. Reyburn, M.D., in Medical Record, October 19, 1901.*

---

**This Journal and Atlanta Semi-weekly Journal one year for $1.00 cash in advance. No checks. Strictly to new subscribers.**

### CLINICAL DATA RELATIVE TO INEBRIETY.

BY SAMUEL BELL, M.D., DETROIT, MICH.

The study of the effect of alcohol upon the human system received a new impetus when recently the N. Y. Academy of Medicine devoted two evenings to the discussion of the subject, together with the critical study of a large number of cases received at Bellevue Hospital by Dr. Charles L. Dana. The latter in his report shows the limitations of the life of the drunkard, also of the periodical inebriate; the maximum capacity of the human body for alcohol; the methods of prevention; the necessity for a special law for the commitment and care of inebriates together with the treatment, temporary and permanent, of this class.

Of 350 cases studied in relation to heredity, he found that drinking habits existed, in one or both parents, in all but ten per cent. In a study of 1,560 cases relative to occupation, he found the following:

| | |
|---|---|
| Professional men | 54 |
| Clerks and salesmen | 239 |
| Tradesmen | 387 |
| Laborers | 589 |
| Waiters | 64 |
| Painters | 64 |
| Liquor dealers | 50 |

In a study of 210 cases, relative to the age at which the drinking habit began, among 30 periodical drinkers two-thirds began drinking before 20 and all began before 30. The investigations show that the habit is formed before maturity.

Dr. Dana also cites some striking examples of besottedness, viz.: A man of 28, a dealer in liquors, drank seven or eight pints of champagne and seven to fifteen glasses of beer daily, for eight years. Then he replaced the champagne with twenty or thirty drinks of whisky. Finally he took up the cigarette habit and this began to finish him. He could no longer stand the drinking, but had an attack of cerebral automatism, and began to develop criminal tendencies.

A man fifty-five years old confessed that he had been drunk twice a day for three years, making about two thousand intoxications. He got home to dinner in a semi-drunken condition from whisky, and went to bed in a similar state from beer.

A man of thirty had been drunk every night for a year and a half.

A man of forty-six had been drunk fortnightly for twenty-five years.

A man of forty had been drunk weekly for twenty years.

A man of forty-three had been drunk a thousand times in fifteen years.

A man of fifty had been getting intoxicated daily for about six months in the year since he was seventeen.

It seems that the capacity for men to get drunk over a thousand times is rare, and that two thousand is the maximum limit in an ordinary inebriate experience.

Drs. Roseburgh of Canada, Crothers of America, Norman Kerr of England, Forel of Switzerland, and other scientific investigators and clinicians have done much to place the study and treatment of inebriety on a scientific basis. The medical superintendents of every hospital for the insane meet with quite a large percentage of what may be termed moral delinquents. They are on the borderland in regard to physical and mental health. In their surroundings may be the breeding-ground of neurotic degeneration; they require hospital or asylum treatment. In another, although less pronounced class, the use of alcohol is preceded by an irritable and exhausted condition of the system, and the sudden removal of spirits is followed by acute delirium. In a third class are periodical drinkers who drink to excess at intervals. In a large percentage of these, investigations have shown that at least one parent has suffered from some form of neurotic degeneration. In a fourth class are often found young men whose surroundings may be conducive to drink. They may be sons of wealthy parents, or, possibly, having inherited plastic or yielding dispositions, they may be the victims of convivial companions. The fifth class take to drink from over-work and general neglect of healthy living. They are often found among business and professional men who are working under high pressure and are generally satisfactory to treat, as they are amenable to treatment and prolonged rest.

The sixth is a not very frequent class, where inebriety is due to an injury of the brain, caused by blows upon the head.

In a seventh class are what are known as Dipsomaniacs. In this class the desire for drink becomes a mania. The patient loses respect for himself, neglects his duty to family and community. The appetite for drink becomes a disease, overpowers every other desire and he is completely enslaved. The treatment of inebriety must be based upon the above divisions and may come under two heads, the ideal and the practical; the former in an institution with good medical attendance and nursing; the latter or practical, home treatment.

In a recent report on heredity presented by Dr. T. D. Crothers are given some interesting facts based on the study of 1,744 cases of inebriety. Of these 1,744 inebrieties, 1,080 had a distinct history of heredity; 430 of these he classes as direct heredity, where the drinking of parents or grandparents reappeared in the children.

The second class he terms indirect heredities, and 224 of the 1,080 were put under this head. They usually were persons whose grandparents, one or two generations back, had been moderate or, on occasions, excessive drinkers.

In the third classification, psychopathic heredity, he places 290. These cases are psychopaths, or persons in whom some defect of brain and nervous system seems to persist for generation after generation. Some of the most brilliant men of the country belong to this class. Paralysis, epilepsy, hysteria and many of the obscure insanities alternate one with another. The sons of most excellent parents may become the most degenerate inebriates, and the children of these degenerate parents become model temperance men. This class furnishes the most interesting, confusing and least understood of all the hereditary cases of inebriety. In the last classification, epileptoid types of heredity, are grouped 49 persons. They were not altogether periodical drinkers, their alcoholic symptoms being always sudden and unexpected. The ancestors of these persons were always noted for sudden, explosive nerve energy. From this class come persons who have periods of "sowing wild oats."

The careful statistics of the Swiss Confederation show that about one-third of the male inmates of insane asylums, one-third of the male suicides, and one-tenth of the men who die at twenty or more, at least in the larger towns of Switzerland, are due to the alcoholic drinking of the victims. Ten families of drunkards produced fifty-seven children, twelve of whom died in infancy, thirty-six were either idiotic, misshapen or had serious nerve trouble; only nine remained normal. Ten sober families produced sixty-one children, of whom five died in infancy and fifty remained normal; only six were either somewhat undeveloped, defective or had St. Vitus' dance.

About the middle of the eighteenth century a drunken woman gave birth to a number of children. A few years ago Professor Pelman ascertained the number and career of her decendants. Among 709 of them 106 were of illegitimate birth, 142 were beggars, 64 were supported by the municipality, 181 were female prostitutes, and 76 had been sentenced for crime, including seven murderers. This melancholy brood had cost the state five million marks. There had been 125 other descendants of whom nothing could be learned. This case demonstrates how much of the wretched element is produced and increased by means of alcohol, and refutes the statement of those who declare that alcoholism exterminates the descendants and so eliminates the degenerates from society.

Treatment such as the physician can render to dipsomaniacs and at the same time allow him or her to have freedom is generally only temporary. This is one reason why a very large per cent. of those who have taken what is popularly known as the "jag cure" relapse; and it is well known to those who have observed this class that every relapse leaves the victim with less will power to resist the temptation to drink—consequently in a much worse condition. For the majority of cases that take treatment require restraint. To produce emesis, cause narcosis and administer remedies to counteract the craving for drink are but temporary expedients. The ideal treatment is institutional, and supervision not for a month, but for a year, is needed—insuring absolute abstinence from spirituous liquor in all its forms—with subsequent supervision for a year longer. The "cures," heralded with great display, have for their basis, strychnine, atrophine and apomorphine, combined with tonics, laxatives and liquid diet, and psychological influence on the patient very ingeniously exercised.

How to carry out proper treatment long enough to be of lasting benefit to the alcoholic patient is often puzzling to physicians and interested friends. The treatment is not compulsory in our State. Inebriates as a class are impulsive, not very appreciative, impatient of proper restraint, and nearly always overestimate their ability to withstand the temptation to drink.—*Detroit Med. Journal.*

---

### THE USE OF ASEPTIC ADHESIVE STRIPS IN THE CLOSURE OF WOUNDS.

Thos. I. Motter, M.D., of Chicago, says: My attention was first called to this method of wound closure by an article by Lilienthal, of New York, who had been using it extensively in his hospital work. The advantages which are offered to the surgeon by such a method, if it can be made to meet the demands of asepsis and strength, are many, the two most prominent being the lessening of wound infection from sutures and the diminishing of the amount of scarring. We all know the great danger of infecting an otherwise clean wound by means of the skin sutures, the sutures themselves acting as a means of ingress to the bacteria, and again by passing through an infected layer of skin which the antiseptic methods of to-day have as yet been unable to reach and render aseptic. This last fact has been so clearly demonstrated that it is only a wonder we do not more often have infection from this means than we really do.

The second advantage to be derived—the lessening of the scarring—is one of almost as great interest as the first. How often we see the results of an operation on the neck, face or hands of a patient, indelibly stamped there by the suture marks. A scar which, if it were a simple line, would not be half so noticeable, is made prominent by the peculiar markings of the stitches that were used. To be able to avoid such a scar would not only be pleasing to the patient, but equally so to the surgeon. By this method such results are possible. There are many other points of advantage of less weight—as the doing away with the removal of sutures—a source of no small dread to many patients; the avoidance of nerve impingement; the ease and rapidity of applying and the ability to reapply at future dressings when no general anesthetic is used. Again, in wounds of the face and hands it renders a great help to the surgeon—as by its use he can thoroughly and evenly approximate the tissue without the use of sutures, a great relief to the patient.

The use of adhesive plaster in this manner has been tried before the present time, but as many times abandoned from its uncleanly nature—making a most undesirable wound. Experimenting has been done in this country and Germany to make an antiseptic adhesive plaster, but up to the present time has failed, owing to the fact that the process of sterilizing the plaster destroyed its adhesive value. The work has had its reward, and to-day we have the desired plaster, both aseptic and adhesive in nature. The plaster is made under the name of "zinc oxide aseptic strips."

I began the use of these strips cautiously, using them first to close wounds of the hands and such minor work. The results there led me to try them in my major work. The first major case I used them on was a unilateral pyosalpinx, which was removed by an abdominal incision. The usual procedure of closure of peritoneum and muscle fascia by catgut was followed, then we had a gaping skin wound an inch in width. This was held together by an assistant, after the surrounding skin surface had been thoroughly dried, and the strips, one-quarter inch in width, applied across the wound at right angles to the incision. The result was perfect—healing by first intention and a scar you could hardly notice. This result led to my adopting it in general surgery wherever possible. I used it in various laparotomies and in appendectomy—in amputations and hernia operations—with all like pleasing results. The cases were all clean and remained so.

When once applied properly it will hold against a great deal of tension, as I fully demonstrated in a case where stretching of the sciatic nerve was done for sciatica. The incision was over the gluteal fold, and when we came to close it we found much tension to overcome. The strips were applied while the wound was held firmly approximated and by careful application the wound was held securely together.

Where drainage is needed they can as readily be used—allowing the gauze or drainage material sufficient room at the lower margin of the wound. This procedure, in a case of appendicitis where a good deal of oozing was present, worked very satisfactorily. The drainage was removed on the third day and the opening approximated by a strip of the zinc oxide plaster.

There were a few points of value gained by experience. My usual procedure was, after the deeper tissues had been united by sutures, then the external surface was thoroughly dried. For this I used alcohol on a sponge. I found better approximation could be made by one-fourth inch strips, applying them about the same distance apart, than with wider strips. This gave a view of the wound at any time. In case one should want to be removed to be applied a little closer it was much easier and not so liable to disturb the rest of the wound as when using the broader strips.

After applying the strips the usual dry dressing of iodoform, aristol or boric acid was applied and the usual pad and dressings followed. In applying the first strip an assistant holds the tissues well together, and applying one end of the strip to the skin surface, the other is carried across the incision, being held tight and applied to the skin surface on that side of the wound. It is best to apply the center strip first and work to either end of your wound. Should any of the strips be put on too loose the first time one end can be readily raised and drawn more snug and again applied. These strips were left on from seven to fourteen days. In case you should have reason to examine the wound you can readily do so by removing the pad and dressings and even further by lifting one end of a strip so as to expose the wound beneath.

Not the least trouble was experienced in removing the strips. The best method is to loosen both ends and gently free them just to the margin of the wound, then taking them both together they can be freed from the wound itself with no danger of pulling it open. In case the strips should stick, a little peroxide of hydrogen applied to them will cause them to be readily and easily removed. My own results have been so satisfactory that I feel free in urging others to try this method—feeling sure they will be gratified by the results obtained.—*Pacific Med. Jour.*

---

### Prostitution and Venereal Disease.

At a meeting of the New York County Medical Society, held on February 25, 1901, Dr. Prince A. Morrow read a paper upon the subject of the prophylaxis of venereal diseases and the medical aspects of the social evil in this city, in which he stated that while the actual number of cases of venereal diseases in a community must always remain an unknown and an unknowable quantity, it has been estimated by a competent and careful authority that fully one-eighth of all human suffering is caused by these diseases or their sequelæ. According to Neisser, gonorrhea is, with the exception, perhaps, of measles, the most universal and widespread of all diseases. Other German authorities have computed that fully three-quarters of the adult male population and one-sixth or more of adult females have contracted gonorrhea. As regards the prevalence of syphilis, Fournier found as the result of careful personal investigation, extending over a number of years, that from 15 per cent. to 23 per cent. of the cases treated at the general hospitals in Paris were of syphilitic origin. In New York city the records of the out-patient departments of five hospitals and dispensaries show that nearly 10 per cent. of those who applied for treatment during the year 1900 were venereal cases. An analysis of statistics shows that the average age at which syphilis is contracted by women is from 18 to 20; by men from 20 to 25.

As a result of Dr. Morrow's paper, a committee of seven was appointed by the Society for the "Study of Measures for the Prophylaxis of Venereal Diseases." This committee, with Dr. Morrow as chairman, sent a circular letter to each of the 4,750 physicians resident in Greater New York, containing a series of questions as to the number and kind of venereal diseases occurring in their private practice during the year 1900. In response to this letter available replies were received from 678 physicians—nearly 20

per cent. ; these reported 15,969 cases of gonorrhea and 7,200 cases of syphilis. Taking this aggregate of 23,196 cases reported by 678 physicians, the committee considered it fair to assume that an equally large proportion of cases occurred in the practice of those who sent no report, and upon this basis of calculation there would be a total of 162,372 cases of gonorrhea and syphilis treated during the past year in private practice in this city.

The figures we have given above amply demonstrate the prevalence of venereal diseases. The gravity of these diseases, their far-reaching significance in relation to the public health and the untold social miseries which follow in their wake we shall not dwell upon.

As to the sources of infection, prostitution is by far the most important. According to the statistics of Dr. Sturgis, there were 30,000 public prostitutes in New York City in 1897, and the number has been recently roughly estimated as between 40,000 and 50,000. We agree with Dr. Morrow that the suppression of this evil is a Utopian idea. It has existed, he tells us, in all ages, and Solomon somewhere cautions the young man against the woman who loitereth on the streets at night. The most severe and drastic measures, carried out under the most despotic authority, have failed to crush it. Almost every conceivable punishment, flogging, branding, shaving the head, banishment and death have been employed in vain. It cannot be annihilated by force. In the existing economic and moral conditions of society it is a necessary evil, not in the sense of being indispensable, but inevitable.

Admitting, then, that it cannot be eradicated, how can it best be controlled? In France, regulation of prostitutes has been employed for over half a century, and the system there followed serves as a type of that practiced in other countries. A special corps of police is employed, every woman in the streets suspected of prostitution is arrested, her name is inscribed on a special register and she is given a card which is an authorization to exercise her trade under certain conditions; the most important obligation is to report at stated intervals for medical examination. When found diseased she is sent to a special hospital and detained until her contagious accidents are cured. This system, it will be seen, rests upon the tripod of police force, medical examination and hospital isolation—the hygienic feature is the medical examination.

Over 200 of those who replied to the letter of inquiry sent out by the committee of the county Medical Society advised segregation as the best measure to limit or prevent the dissemination of venereal diseases, but the committee in its report did not deem it wise to advise this, as it would imply a legal recognition or sanction of the evil. The committee states that from a common-sense as well as a humanitarian point of view, it is evident that these unfortunates must be permitted a habitation. They cannot be forced into the river and drowned, as 800 of them were at one time under the edict of a French emperor. They cannot be driven forth into the hills and fields, denied food and shelter and left to perish as was done at a later period in Edinburgh. The brutal methods carried out with savage energy which characterized former crusades against these unfortunates are opposed to the spirit of the age in which we live—certainly they find no place in the counsels of enlightened sanitary science. What then shall be done with them? The most feasible plan appears to be to compel all prostitutes to inhabit houses by themselves. Immoral women should not be allowed to dwell in the same house with moral families. This domiciliary separation should be absolute and complete.

Among other measures suggested to the committee was regulation by the Board of Health, the compulsory report of such cases to the health department, and isolation; penalizing the transmission of syphilis was also recommended.

The most important measure, in our opinion, in the prophylaxis of venereal diseases is the education and moral development of the masses. When prostitution is no longer profitable, it will soon die out, but as long as the charms of a woman's body have a marketable value, so long will women be found to prostitute themselves for that purpose. Perhaps, in the milennium that is coming someone will succeed in reforming all the young men—and the old ones, too. Then, as the demand for prostitutes ceases, the supply will cease also, and these women will seek more lucrative employment.

### THE SURGEONS OF THE CONFEDERATE ARMY AND NAVY.

BY E. D. NEWTON, M.D., MILLEDGEVILLE, GA.

The medical officers of the army and navy of the Confederacy were noted for their ability, skill and unselfish patriotism. What was true of the medical officers of the army of Northern Virginia (Lee's army) was also true of the entire medical corps of the Confederate army and navy. Dr. LaFayette Guild, surgeon and medical director of the army of Northern Virginia, writes as follows in a letter to Dr. Samuel Preston Moore, Surgeon General, dated Fredericksburg, Va., May 22, 1863:

"Nearly all of the members of our corps with this army have undergone a careful examination before the medical board, and I can truthfully assert that as a body of professional gentlemen they will compare favorably with any other similar organization upon this continent. However, they need no encomiums from me to establish their claims to zeal, efficiency, intelligence and patriotism in the discharge of their responsible duties. Their conduct on so many bloody battlefields has secured to them an enviable reputation and has elicited deserved praise from all who have witnessed their noble self-sacrifice during and after a battle."

Anxious to rescue from oblivion the history of the medical departments of the Confederate States army and navy in 1873, I opened a correspondence with a number of ex-Confederate surgeons in Georgia and the South, and in 1874, May 20th, a meeting was held in the city of Atlanta, and the Association of Medical Officers of the Confederate Army and Navy was organized, its object being the collection and preservation of the medical and surgical records and statistics of the late Confederate army and navy, and the collection and publication of such scientific facts as may be useful to the association and the medical profession, the preparation and publication of biographical notices of deceased members of the late medical staff and the cultivation of social and friendly intercourse. Dr. S. H. Stout was elected Temporary Chairman, and Dr. Charles Pinckney, Temporary Secretary.

Permanent organization was effected by the election of late Surgeon General S. P. Moore, President; Surgeon Henry F. Campbell,

Vice-President at large; Vice Presidents, Dr. Joseph E. Claggett, Maryland; Dr. Hunter McGuire, Virginia; Dr. S. S. Salchwell, North Carolina; Dr. A. M. Talley, South Carolina; Dr. W. H. Westmoreland, Georgia; Dr. E. A. Holt, Florida; Dr. C. J. Clarke, Alabama; Dr. S. V. D. Hill, Mississippi; Dr. E. S. Drew, Louisiana; Dr. S. H. Stout, Secretary, Atlanta; Dr. Charles Pinckney, Assistant Secretary, Atlanta; Dr. E. D. Newton, Treasurer.

By invitation of Dr. Hunter McGuire, Richmond, Va., was selected as the place of the next annual meeting, October 18th, 1875.

The Richmond meeting, as well as the Atlanta meeting, was a marked success socially. Since 1875, though efforts have been made by a few enthusiastic comrades, but little has been accomplished to carry out the objects of our association. Let us hope that we may make more progress at Memphis, and that we may draw an inspiration from the presence of the rank and file of our gallant boys in gray.

As a matter of interest we may mention that the medical department of the Confederate States of America was organized in Montgomery, Ala., with the appointment of Dr. D. C. DeLeon, of Alabama, a "resigned" surgeon of the United States army, as Surgeon General. In the summer of 1861 Dr. DeLeon was succeeded by Dr. Samuel Preston Moore, of South Carolina, and late surgeon United States army. Dr. Moore held his position during the remainder of the war.

I am sadly reminded that the surgeons of the Confederate States army and navy are one by one "passing away," by the fact that I am the only survivor of the immediate staff of Dr. L. Guild, Medical Director, Lee's Army, and that Dr. Joseph E. Claggett, the surgeon in charge of "the Receiving and Forwarding Hospital" of the Army and Navy of Virginia, alone survives all of his own staff.

Surgeon Hunter McGuire, medical director of the Second Corps (Jackson's), is dead. So are Dr. J. S. Cullen, surgeon and medical director, First Corps (Longstreet's), and Surgeon J. B. Fontaine, medical director, Cavalry Corps. I do not know whether J. W. Powell, of the Third Corps (A. P. Hill's), is alive or not.—*Southern Practitioner.*

### EXTINCTION OF GYNECOLOGY.

According to that veteran in the ranks of the American Medical Association, Dr. Henry O. Marcy, of Boston, gynecologists are rapidly becoming the specialists in abdominal surgery, and consequenty gynecology as a specialty must soon disappear. To us that seems like queer logic. It is, however, the natural result of a constant training along wholly operative lines. It seems next to impossible for a man who once does much cutting to ever again see a case of disorder in any other light than that of a candidate for the knife. It seems to us deplorable that such is the case. It would seem that the large number of mutilated and surgically patched up women who constantly present themselves ought to prove a warning against the miscellaneous surgery of the time, but they are slow in making their influence felt. Nine-tenths of the suffering women are far better off in the hands of an intelligent practitioner than in those of the school of "abdominal surgery." The former gives the patient the benefit of an interrogation of all of her functions, even if not fully understood, while the later so constantly sees absolutely nothing but a chance to change her anatomical integrity.

The true gynecologist, and the safe one, is he who has a training in general medicine, and especially along neurolgic lines as well as in pelvic and abdominal surgery, and who never fails to give his patient the benefit of a systematic study of her sex in relation to her manifestations of disorder. By so doing, and if he is not dazzled by scintillations of modern surgical genius, he will often be able to see wherein measures of calmer type and dissimilar character are capable of accomplishing a better, more enduring, and in all respects more satisfactory purpose.

No, the true gynecologist is not going. He is preparing to drive out usurpers of the title, study closely the natural succession of inflence and effects, include the act of parturition within his sphere for purposes of continuity of observation, and control the development and progress of local disorders by means that may insure the greatest degree of conservatism. His dominant thought will be of *salvation* and *not extirpation.* He will not go into a fit of hysterics at the bare mention of electricity, but will investigate and understand its action. He will first give its claims the serious attention they deserve and be able to talk advisably. He will not fall in a faint at the suggestion of "local application" and massage until he learns from actual experience that they are wanting in remedial influence; and will not collapse with chagrin at the idea that a woman's pelvic organs were not designed for artistic modeling to suit the fancy of each successive manipulator. He will realize that woman's reproductive organs have no isolated pathology and that their functions are but a part of "one stupendous whole," and that their relationship to the whole economy justly argue a careful attention to mutual influences in order to gain the most desirable results, an attention that will observe subtle reactions in themselves sufficient to overcome disorders under proper guidance.

The gynecologist of the future will be more of a scientist and less of an artisan. He will study the suceessive steps of cause and effect and overcome aberration by exciting antagonistic tissue changes along lines compatible with the maintenance of functional integrity. The man whose activity centers in the idea of revolution rather than evolution will never become a successful creator, or re-creator in the organic world. His mechanical genius should find its place in the sphere of physics and not in that of chemistry.

The gynecologist of the future will be found doing all he can to eliminate the surgical element of his specialty, and not waste his time in devising new methods of unnecessary mutilation. When he does operate it will be because he must in his ignorance do so, and not because of preference. He will do so merely as an expedient to which lack of knowledge has driven him, and the necessity for which he constantly hopes in time to eliminate with the aid of physiological chemistry and its adjunct sciences.—*Peoria Medical Journal.*

---

### ALCOHOLISM AMONG WOMEN.

There is a growing tendency among women to the indulgence in alcoholic beverages. Among the wealthier and higher classes the habit has become almost universal; and this pernicious example has rapidly extended through the various social substrata, for it is human nature to imitate the actions of those who are better favored, and a bad example, like bad news, travels fast.

The exhausting effects of the demands of society upon its devotees creates a desire for a stimulant, and hence the ever-present and generous punch-bowl is often the most popular feature of the social event. It adds a sparkle to the eye, color to the cheek, and a zest to the spirit of the maid and matron, a delightful feeling of *bién aise*, and its alluring seductiveness bids its partakers to return again and again. Many a young woman, ignorant ot the taste and effects of alcoholic stimulants, has first been brought to a knowledge of its allurements at the social gathering around the punch-bowl. Once a taste, it becomes a difficult matter for women to refuse, should she desire, when offered to her in a new and tempting form, until soon an alcoholic stimulant is essential to her neurotic constitution and the daily potion becomes a fixed habit.

A spirit of recklessness in young women to imitate in secret this vice of the male sex is not infrequently a cause for the beginning of alcoholic indulgence and for the continuance of which opportunities are easily obtainable. So prevalent indeed has it become that there are but few young women of the better classes in the cities who are not familiar with the taste and effects of alcoholic stimulants, and too frequently the so-called attacks of nervous prostration among women are but the after-effects of alcoholic indulgences.

Among the less favored classes the causes that obtain among the wealthy operate necessarily to a smaller extent, but here other conditions act no less powerfully as causative agencies in the formation of the habit. In England the habit of alcoholic indulgence among women of the lower classes is much greater than in America. It is stated that in the epidemic of arsenical poisoning, which occurred last winter in Manchester, England, and its vicinity among beer drinkers, the majority of the victims were women. Dr. Heywood Smith, of London, gives as reasons for alcoholism among women, the increasing independence of women—a liberty which some of them interpret as license for self-indulgence in accordance with their inclinations. In the struggle for life which this independence engenders, there is often the element of failure or overstrain, and women, too weak in many instances to bear the strain, resort to stimulants. The cares, worries and anxieties regarding the home and children, and when especially to this may be added a husband's neglect or the brutality of a husband who drinks, often cause women to seek forgetfulness in the stimulating effects of some alcoholic beverage.

Whatever be the cause, women seldom have the power to resist its temptation when once they become a victim to its use, but woman-like, plunge headlong to excess. In the reformatories for criminal inebriates in England there are five times as many women as men, according to Heywood Smith.

Men drink in company for sociability sake, but women rather in secret, which makes detection difficult at an early period. Often the family physician is called to relieve a distressed condition of the head or digestive organs, or possibly a sudden attack of nervousness in some of the female members of his most respectable families, and in response to the question of the fair patient, diplomatically gives some impossible condition as the probable cause.

The life-history of the female inebriate is identical with that of the male, the only difference being that with a more rapid loss of self-control she passes quickly to the depths of degradation and thus earlier reaches the end.—*Courier of Medicine.*

---

### INSANITY IN WOMEN FROM THE GYNECOLOGICAL AND OBSTETRICAL POINT OF VIEW.

From a careful study of a large number of recent articles by writers of great knowledge on the subject of the relation of pelvic disease to the production of insanity, added to his own experience, A. Lapthorn Smith believes the following conclusions to be justifiable:

1. Insanity is not hereditary as is generally supposed, but it certainly is sometimes contagious.

2. Insanity in the majority of cases is not due to organic disease of the brain, but to functional disorders of its circulation and of its circulating fluid.

3. In many cases, in women, the disorder of the brain's circulation is caused by reflex irritation carried by the sympathetic from the pelvic organs (as by retroversion of the uterus, cirrhotic ovaries, fibroid tumor, etc.).

4. In other cases it is the fluid circulating in the brain which is at fault; in some it is too poor quality, because the digestive apparatus is interfered with by reflex irritation ot the sympathetic, due to lacerated cervix, endometritis, etc.

5. In a lesser number of cases the brain is prevented from working because the blood is badly oxygenated, or loaded with uric acid, urea or other poison.

6. Hundreds of cases are now on record of insanity being cured by removal of the cause, the greatest number of mental cures having followed ventro-fixation and shortening of the round ligaments, for the removal of retro-displacements, while many others have followed the ablation of fibroids, cirrhotic ovaries, the repair of lacerated cervices, and even simple curetting.

7. Such being the case, it is the duty of the family physician to examine carefully every woman in his practice who becomes insane, or to have her examined by a gynecologist; and, if any pelvic disease is discovered, it should be remedied.

8. It is the duty of every medical superintendent of an insane asylum to have a systematic examination of every female patient made (preferably under anesthesia), so that unsuspected sources of irritation of the sympathetic situated in the pelvis may be removed. In one asylum alone this course has resulted in improvement in 60 per cent. and recovery, mentally, of 42 per cent. of those operated upon, although the pelvic troubles had existed for from six to sixteen years.

9. If anything is done it must be done thoroughly, as several cases have been reported where no benefit resulted until a second and more complete operation was performed.

10. In view of the number of women who become insane from uremia, more care should be exercised by practitioners in preventing this condition. All Protestant physicians should, with the advice and approval of one or two colleagues, empty the uterus before the kidneys become permanently damaged. Catholic physicians are not allowed by their church to sacrifice the ovum in order to save the mother, but they may do much by internal medication to prevent the dire results now so often noted.—*Amer. Jour. of Surgery and Gyn.*

---

### COUGHS.

All coughs are either moist or dry. A moist cough is nearly always paroxysmal; expectoration is usually most abundant in the morning. This cough, like all others, is nearly or quite suppressed toward the fatal end of most grave diseases, owing to carbon dioxide narcosis.

Anatomically, most coughs are either pulmonary or bronchial. The pulmonary class is marked by more or less percussion dullness, and by double subcrepitant and inspiratory crepitant râles or bronchial breathing. The bronchial class is marked by soreness, oppression, pain and irritation in the upper sternal region, and by moist double râles.

A dry cough is usually short, sharp and hacking, though sometimes paroxysmal. Reflex forms are generally quickly relieved by treating a local cause, or they may be produced artificially by irritation of the affected locality. There is inability to cough in bulbar paralysis and extensive destruction of the larynx. A dry, pulmonary cough is accompanied by broncho vesicular or bronchial breathing and impaired resonance.

Dry bronchial coughs are tight and harsh, with sonorous and sabilant râles.

Laryngeal coughs are hoarse, harsh, deep and rough, with altered voice and laryngeal pricking, burning and soreness, and a constant desire to clear the throat.

The pharangeal cough is accompanied by a pricking feeling in the throat or feeling of fulness.

Nasal coughs are marked by local signs and by "hawking" down mucus from the posterior nares.

The faucial cough is usually worse on lying down, and is attended by a tickling in the throat.

The oral cough is due to irritation of tongue or teeth.

Aural coughs are due to irritation of the auriculo-temporal branch of the fifth nerve, and may be accompanied by considerable expectoration.

Pleuritic coughs are generally painful, with quick and painful breathing, and often friction murmurs or flatness.

Pressure on the respiratory tract by tumors or pseudo-tumors, excites a cough, which is laryngeal in character.

Visceral disease is a rare cause of cough, and diagnosis should be made by strict exclusion.

A uterine cough is hacking, very painful and trying, and repeated two or three times in succession. It is excited by the least irritation.

Nervous coughs deserve considerable attention. They are periodic or paroxysmal, usually high-toned, quite variable, slight or prolonged and painful. Two important general characteristics are that they disappear entirely during sleep, and are accompanied by no secretion whatever. On auscultation there are sometimes wheezing, rattling, scraping sounds, and there may be spasms, convulsions or aphonia.—*Denver Med. Times.*

---

### TREATMENT OF CHRONIC ENDOMETRITIS IN GENERAL PRACTICE.

Menge (*Medical Press,* July 31, 1901) believes the reason why this disease is so much neglected by the general practitioner lies in the want of technical dexterity. The writer was led by this conviction to search out a method of treatment that would be equally useful in all forms of the disease, that would be as free from danger as possible, and that would demand the least possible amount of manual procedures. The treatment should admit of being carried out rapidly in the consulting-room, and without previous dilatation of the cervix. After touching upon the usual methods of treatment, he proceeded to describe that practiced by himself, which in the course of years has proved to answer all the requirements both on the side of the general practitioner and of the patient. A solution of formalin, from 30 to 50 per cent. in strength, has proved a useful and non-painful intra-uterine application. For the purpose of applying such a solution he has had constructed a smooth, hardrubber sound, thinner toward the end, armed with a thin layer of absorbent cotton to a distance of forty centimeters, and kept for use in a cylinder filled with the formalin solution. The caustic is never applied after a bimanual examination, but always some days afterward. It is applied in the following way: A freshly boiled Trelat or Neugebauer speculum is introduced into the vagina. After the os is found it is wiped with a pledget soaked in perchloride solution. The anterior lip of the uterus is now hooked for the purpose of steadying it, not to draw it down. Two or three formalin sounds are now introduced one after the other and passed in all directions, so that the bottom of all the folds of mucuous surface shall be reached. A strip of gauze is now introduced for the purpose of draining the cavity of superfluous fluid. In the early part of the treatment it is best to wait until the mucous surface is renewed before applying the caustic the second time. Later on, however, it may be applied at intervals of a week.

The formalin caustic has a beneficial effect in chronic endometritis whether following labor or abortion, provided that no placental remains are *in utero*. These should not be burned off with the caustic, but after one or two applications they should be removed with the curette. Post-gonorrheal endometritis may often be cured in this way after very few applications, provided general treatment be kept up at the same time. If, on the other hand, the endometritis is due to general disturbance, but little is to be expected from local treatment. There must be a rational combination of general and local treatment in some cases. He mentions the contra-indication to the treatment, which will be obvious to any one who gives the matter a thought. When there is malignant change in endometrium the treatment is inadvisable.—*Med. Age.*

---

### TYPOGRAPHICAL ERRORS.

THE editor of the *Journal of the American Medical Association* is to be congratulated. He knows how to acknowledge a typographical error, and to get out of it neatly. This is a rare virtue and much more commendable than the cry of the educational Pharisee, who claims that he makes no errors and who thanks God that he is not as other men are. A paper devoted to "The Cause, Prevention and Cure of Poverty and Pregnancy" will doubtless excite curiosity until the world reads the editor's graceful announcement that the author meant "Poverty and Degeneracy." This is not quite so bad an error as the one that occurred in an old edition of the Bible (a copy of which is preserved in the Bodleian Library at Oxford), in which the little word *not* was left out of the Seventh Commandment. The few rare copies that were preserved when the edition was suppressed, now command a premium among Bibliophiles. Perhaps this will yet be so in the case of our contemporary.

The editor who proclaims that he never makes any typographical blunders is on dangerous ground. With his own types he is likely to be condemned. Thus an editor who makes a specialty of being typographically immaculate, recently made one of his contributors speak of "a hemi-anesthesia of the conjunctiva of the arms and body." This was a great stretch for the conjunctiva, but as the editor is an eye-specialist the world probably accepted the statement as authentic. Again, an author recently wrote a paper on Spinal Cocainization, but the title was printed in large letters by this same editor as *Special* Cocainization. Such an error is not strictly typographical—it is, rather, an editor's blunder, for an editor should see at a glance that such a title is a misnomer. The *Lancet* recently spoke of "a sour correspondent" (as some of them really are) when it only meant to say "as our correspondent."

But editors have good precedent in this field. Disraeli tells us (*Curiosities of Literature*, Vol. 1.) that when Pope Sixtus V. issued his edition of the Vulgate he prefixed to the first volume his bull excommunicating all printers who should make any alterations in the text; but the work was found to be so full of errors (although the Pope himself had corrected the proof) that scraps of paper containing the corrections had to be pasted over the errata. This should be a warning to our infallible editors who proclaim constantly that they never make a mistake.

The great trouble that can come from a little misplaced comma was illustrated once in a small New England seaport. A clergyman read a notice from his pulpit that "Captain Jones, going to sea his wife, desires the prayers of the congregation." And so may all editors, going to print, desire the forbearance of their readers.—*Dietetic and Hygienic Gazette.*

---

### To Control Hemorrhage in Operations Upon the Head and Neck.

The partial or complete obliteration of the common carotid arteries for a temporary period is the basic principle of a method advanced for the control of hemorrhage in operations upon the head and neck.

The instrument employed consists of two parallel clamp blades shielded by rubber and adjusted by a set screw. The effects on the blood pressure and respiration comprised the main physiological factors of the investigation. The blood-pressure curve in all cases rose to a height corresponding as to whether one or both carotids were clamped. Physiological compensation, however, occurred soon after, the blood pressure curve thereby again regaining its normal level. Cardiac inhibition, due to vagal manipulation, was noted in many cases. Its results were not attended with serious consequences, and experimentally as well as clinically this disturbance is effectually eliminated by atropine administered some time previous to the operation. Respiration for the most part was unaccompanied by any marked changes, a slight fall being noted in many cases. Changes in the arterial coats varied according to the amount of pressure applied, and the length of time during that such pressure had been continued. The lumen of the vessel in all cases showed an oval outline at the point of application, especially in those cases where the clamp had been too tightly adjusted or had been maintained for a continuous period of several hours or days. Clamping the arteries for fifteen to twenty minutes gave otherwise no change. When continued for a longer period, one hour or so, alterations of the intima were noted, such as separation and massing together of the endothelial cells. Media and adventitia also bore evidence of disturbances in their component elements.

For the clinical application of this method, caution against too forcible adjustment is advised. Mere approximation of the arterial walls, not compression of the same, is all that is required. Clinically, this method has been efficiently employed during the past few years, and had been found to effectually control arterial hemorrhage. The great number of operations in which this method has been applied comprises the various tumors of the face, neck and nose, parotid glands, excision of the tongue, resection of the jaw, etc.—in short, all operations on the head, neck and face. —*Dr. Geo. W. Crile, of Cleveland.*

---

### A SPECIFIC FOR CHRONIC RHEUMATISM.

How few are the practitioners of medicine who have not experienced failure after failure in relieving the symptoms of chronic rheumatism and its congeners, myalgia, sciatica, etc., by the usually recommended forms of treatment?

The writer has had the same experience, and after many failures with the salicylates, iodides, salol, colchicum, counter-irritants, etc., devised the following prescription:

| | | |
|---|---|---|
| Ext. xanthoxylini frax fl. | | ℥i–ii |
| Ext asclepiadis cornuti fl.<br>Ext. solani dulcamaræ fl. | aa | ℥ss–i |
| Ext. taraxaci densleonis fl. | | ℥ii |
| Spts. frumenti q. s. ad | | ℥viii |

M. S. ℥ss t. i. d. after meals.

This has proven as much of a specific for these cases as quinine does for malaria, or mercury for syphilis. This may appear a broad statement to assert, but after two years' use, in which many cases have been treated, there is not a single failure to record. This may sound like a patent medicine testimonial, but it is not, and if my experience has not been an unique one, I think others who try the above prescription will bear out my statements.

Do not understand me to say that the mixture will cure those cases wherein we have to deal with anchylosed joints, contractures and the like, but in those incipient, though well-marked, cases where the patient complains of stiff joints, lumbago and distressing pains, I have not a failure to record. In one case of inflammatory rheumatism of six weeks' standing, when the classical remedies had all been administered without benefit, two bottles of the above mixture gave rapid and permanent relief.

Were it not that the readers of the *Lancet-Clinic* might accuse me of encephalic softening, I should like to report several other instances in which the remedy acted almost like magic, but I shall refrain, trusting that some one who has a larger *clientèle* among this class of suffers will give the question a fair trial and report results.—*P. C. Layne, M.D., Proctorville, O., in Lancet-Clinic.*

---

### TETANUS RESULTING FROM VACCINATION.

We had scarcely recovered from the surprise of tetanus following the injection of anti-diphtheritic serum in St. Louis, when it was announced that tetanus had followed vaccination in New Jersey. Both of these accidents are to be deplored, and especially the latter, because of the comparative harmlessness of vaccination and its prevention of so loathsome a disease as smallpox, which at present is epidemic in many parts of the country. Further, we have to contend with the anti-vaccination sect, which I regret to say is made up in part of members of the medical profession. It seems to be questionable as to whether the tetanic germs were in the vaccine virus or whether they found their way into the wounds from other sources, as from the clothing and the want of proper cleanliness at the time of vaccination. It is certainly a great calamity that tetanus should follow vaccination, for there is nothing more certain in the whole history of medicine than that proper vaccination is a preventive of smallpox, and this accident will place many barriers in the way of preventing and stamping out this loathsome disease. However, if it can be ascertained that the tetanic germ was not in the virus and that it did find its way into the wound through some other source, then the normal equilibrium of public sentiment concerning vaccination may be regained; otherwise it will be a long time before the timidty of the people will allow them to consent to be vaccinated. Of course the anti-vaccination set will double their energies and make a desperate effort to prevent anybody from being vaccinated, and will doubtless find many adherents.—*American Practitioner and News.*

### THE THERAPEUTICS OF WHOOPING COUGH.

T. J. Mays advocates the application of counter irritants over the region of the pneumo-gastric nerves in the neck, and states that, in his experience, this method is the only one which leads to any noted amelioration of symptoms. His method is thus described:

Trace the pulsating carotid artery from behind the angle of the lower jaw to the clavicle on both sides of the neck. This will be a landmark for finding the pneumo-gastric nerves, which lie in close proximity and slightly behind the carotids. Gentle massage and kneading the region of the neck, every hour or two, yield beneficial effects in many cases of this disease. The application of a strip of mustard plaster, about two inches wide, from the angle of the lower jaw to the clavicles on each side of the neck two or three times a day, until the effects of the mustard are evident, is almost sure to cause amelioration of the spasmodic cough. Equal parts of gum camphor, chloral hydrate and menthol applied over this region are also very useful. Painting the same area with tincture of iodine, twice a day, until irritation of the skin is produced, is a beneficial procedure. Finally, in very stubborn cases the hypodermic injection of silver nitrate over the vagi must be resorted to in accordance with the following plan: Lift the skin over the vagus between the thumb and the forefinger of the left hand, introduce the hypodermic needle just under the elevated skin, and inject five minims of two-and-a-half per cent. solution of cocaine hydrochloride. Detach the syringe from the needle and allow the latter to remain in the puncture. Wash out the syringe with water, draw a two-and-a-half per cent. solution of silver nitrate into the syringe, attach the latter to the needle, and throw in from three to six minims of the silver solution.—*N. Y. Medical Journal.*

---

### THE PREVENTION OF OPHTHALMIA NEONATORUM.

In an article on the prevention of ophthalmia neonatorum, Dr. Lucien Howe, of Buffalo (*Philadelphia Medical Journal*, January 18, 1902), whose name is so prominently identified with this subject, urges the enactment of laws which will make it compulsory upon the practitioner to adopt some form of prophylaxis against this disease, which is responsible for so many cases of blindness. He cites statistics by Kostling, showing that in 17,000 births where no prophylactic treatment had been employed some trace of ophthalmia developed in over nine per cent., whereas in 24,000 children treated by the Crede method the number who developed the disease was only one-half of one per cent. The Crede method, however, has the disadvantage of always producing some pain and usually more or less conjunctivitis, while in a few instances it has given rise to corneal ulceration. According to the statistics of Piotrowski, in 1,030 children treated with a strong solution of boric acid and a ten per cent. solution of protargol, not a single case of ophthalmia occurred, while slight catarrhal conjunctivitis was observed in only 1.2 per cent. Aside from the numerous favorable reports on the value of protargol as a prophylactic against this affection by European authors, the drug is preferred for this purpose by many ophthalmologists in this country, including Drs. Alt, Peck, Cheney, Fox, Hotz, Zimmermann, Converse and Todd. In commenting upon Dr. Howe's paper, the *Philadelphia Medical Journal* remarks editorially: "If we cannot reach the fons origo of ophthalmia neonatorum, we can at least save the offspring from a life of darkness, and protect the community from a source of burden and expense. That this can to an enormous extent be accomplished by prophylactic instillation need hardly be repeated, and its negligence constitutes a sin of omission that deserves commensurate punishment. The enactment of such a law is feasible, its interpretation obvious, and its enforcement not difficult, provided the accoucheur receives the intelligent support of an intelligently instructed community."

---

### PROLAPSUS OF THE RECTUM IN CHILDREN.

Custom says (*Annals of Surgery*) that all cases of true prolapse of the rectum will show a tumor projecting out of the anus. At the base of the tumor will usually be found a sulcus between the mucous membrane of the prolapsed gut and the skin of the anal orifice. In almost all cases the lumen of the gut may be seen in the center of the tumor. There may be inclusion of the peritoneum in cases of prolapsus, but this occurrence is fortunately rarely met with. One of the most important causes of prolapse is infection, whether produced by a retention of the feces or by diarrhea. This applies, of course, to young children. In older children and adults the prolapsus is often due to the presence of a polypus, an ulcer, hemorrhoids, or some other lesion of the rectum. The judicious use of a rubber rectal plug to keep the prolapse reduced, cleanliness, and tonic treatment with the use of strychnin, will probably give the best of results. Polypi, hemorrhoids, or other local lesions will require a surgeon's care; irreducible or constricted prolapsus will have to be resected. Mikulicz first cuts through the outer intestinal tube in its anterior circumference, catching up each bleeding vessel as it appears and ligating it with fine catgut. As soon as the peritoneal pouch has been opened its interior is examined for the presence of small intestine. The peritoneal cavity is then closed by a running suture. The anterior aspect of the internal intestinal tube is cut through little by little until it is opened, and then both intestinal tubes are united by deep silk sutures to the entire line of the incision. The posterior surface of the prolapsus is treated in absolutely the same way, both intestinal ends being united by means of silk sutures. He simply covers the line of sutures with iodoform, places a strip of iodoform gauze over this, and then a wood wool cushion. Daily irrigation with a mild antiseptic solution should be used, opium given internally for a week, and the patient kept upon a diet leaving little intestinal residue. The results of operation are usually excellent. —*The American Journal of Obstetrics.*

---

### RATIONAL TREATMENT OF GOUT.

Although there is a good deal of analogy between gout and acute rheumatism, it is generally admitted by modern physicians that uric acid constitutes the active agency of gout, while lactic acid is inherent to rheumatism.

Referring to the diagnosis of gout only, the following symptoms are worth mentioning: Gastritis, enteritis, hemorrhoids, migraine, neugralgias, sciatica, lumbago, asthma, ocular inflammations, varicose veins, nephritis, gravel, and we may add pulmonary catarrh in the aged. As can be readily understood, gout is in the main an affection of the stomach, liver, pancreas and kidneys. Hence the necessity of instituting at once a treatment regulating the functions of digestion and assimilation. This is readily accomplished through the persistent and systematic administration of Kutnow's powder, two or three times a day, an hour before meals. This preparation corrects the acidity of the stomach, and regulates the liver, while acting on the kidneys. Special attention must be paid also to the diet, without losing sight, however, of the fact that it is always unwise to starve the patient to death. Malt liquors are to be avoided, but red wine at meals is beneficial. The milk diet, so much advocated, is also objectionable in many cases. Under the pretense that gout is eminently a disease of the well-to-do, meat is generally forbidden; yet meat well cooked can do no particular harm, if sparingly used. The administration of Kutnow's powder will permit to feed the patient, providing proper care is taken of the idyosincrasies.

The pain in the joints is speedily relieved with crude petroleum briskly applied to the affected parts. When practicable, hot air baths (Tallerman's method) may be administered. As to the preparations of salicylic acid, among them salicylate of sodium, although they give relief, they are apt to play havoc with the patient, particularly when there is interstitial nephritis. We may keep in mind, also, that the timely administration of Kutnow's powder is an admirable preventive.

---

### SURGERY IN THE PRESENCE OF SUGAR IN THE URINE.

Fisk (*Annals of Surgery*) reports a number of cases in which he operated successfully in the presence of sugar in the urine. He reviews the literature of the subject and draws the following conclusions: The presence of glycosuria in those individuals who may have surgical diseases does not in itself constitute an absolute contraindication to any and all surgical relief. Very great judgment must be exercised in the selection of cases, in the determination of the kind and extent of the operation to be performed, and the strictest surgical asepsis must be rigidly enforced throughout. Infection, when it occurs, is from without, and is the result of an error in technique; it thus happens the constitutional symptoms become more serious and out of all proportion to the local, generally ending in death. When infection does not occur the operative wounds heal kindly, but slowly, especially granulating wounds. The vascularity of the tissues must be interfered with as little as possible, so that every operation should be planned with this object in mind. This is particularly so in gangrene of the extremities, in which the statistics of Heidenheim, Kuster, and Smith and Durham show most conclusively the necessity of high amputations in these conditions. He is of the opinion that it is better to cut down upon and ligate the artery in gangrene of the extremities rather than to attempt the bloodless amputation by means of the Esmarch band, because of the possible harm to the tissues, especially the blood-vessels, whose vitality is not the best. —*American Journal Medical Sciences.*

---

### CONDITION OF THE KIDNEYS WITH REFERENCE TO THE EMPLOYMENT OF DIURETICS.

A. R. Elliott (*Med. News*, vol. 79, no. 6) concludes a valuable and timely article thusly:

1. Except in the case of the irritant-epithelial diuretics (turpentine, cantharides, etc.), the entire class of diuretics may be said to exert their effect upon the urine by acting indirectly through the circulation.

2. Owing to the necessity for sparing the kidneys all irritation, drugs given for diuretic purposes should act indirectly rather than directly; consequently the secretory diuretics are contra-indicated in irritative and inflammatory renal conditions.

3. In functional urinary disorders diuretics are mainly useful to overcome concentration and hyperacidity of the urine. To accomplish this, simple dilutents and salines are best adapted.

4. In acute nephritis saline diuretics are permissible throughout the entire course of the disease, and exert a beneficial influence by increasing elimination and clearing the tubules of inflammatory debris. Subcutaneous saline infusion constitutes our most powerful eliminant in desperate cases.

5. In chronic nephritis the cardio-vascular diuretics are most useful, owing to the fact that oliguria and dropsy are usually the result of circulatory failure. The dropsy under such circumstances, being of cardiac origin, may be benefited by cardio-vascular stimulants, provided the kidneys are not too badly damaged.

6. Dropsy of purely renal origin is not amenable to favorable influence by diuretics.

7. Although the morbid process in the kidneys may furnish us with our primary inspiration to diuretic medication, in most cases that determines the choice of an agent.

---

### STRICTURE OF THE ESOPHAGUS.

While the diagnosis of this condition is usually to be easily and satisfactorily made by means of the esophageal bougie, the use of this instrument is sometimes inadmissible, owing to suspected aneurism of the aorta, hematemesis, the weakness or disinclination of the patient. Under such conditions a method described by Holzknecht (*Deutsch. med. Woch.*, September 6, 1900) is likely to be of great value, as it gives accurate information regarding the presence, locality, caliber, and length of a possible stricture in a simple and painless way that is free from all risk. On illumination of the chest by means of an X-ray tube placed below the right shoulder and with the fluorescent screen arranged so that the line of sight runs from the left behind, forward and to the right, the esophagus is visible as a light streak occuping the position between the dark masses of the heart and the vertebral column. On giving the patient a small quantity of water holding in suspension 15–30 grs. of subnitrate of bismuth, if a stricture be present the fluid will be arrested at this point and a precipitation of the metallic powder takes place, which will throw an appreciable shadow on the screen. The test is mainly useful in stenoses of small caliber, and if it prove negative, the bismuth should be enclosed in a capsule, and the process of deglutition observed as before. If this, too, fails, it may be well to repeat the experiment, having preceded the administration of the capsule by the giving of a small morsel of bread, which is sure to be arrested and so impede the progress of the test object.—*Medical News.*

---

### SOME OBSTINATE BLADDER CASES.

BY GEORGE W. HOPKINS, M.D., CLEVELAND, OHIO.

John C., aged 31. Occupation, patrolman. Following exposure, patient experienced bladder symptoms as follows:

Frequent urination, tenesmus, hypogastric pain and a temperature of 101.4 degrees.

The urine was scanty, turbid and loaded with mucus.

Diagnosis: Acute Cystitis.

Treatment consisted of rest in bed, restricted diet, anodynes for the tenesmus, diluent and alkaline drinks.

The acute symptoms promptly subsided, but the urine continued abnormal despite the general measures employed and the internal administration of urinary antiseptics.

Irrigations with Boric Acid solutions of varying strength proved unsatisfactory, as did also solutions of Potassium Permanganate and Silver Nitrate similarly applied.

A twenty per cent. solution of Glyco Thymoline was then substituted for irrigation, and the improvement was marked and continuous until recovery was perfect.

Harry R., aged 43. Occupation, bookkeeper. Had a history of bladder trouble of several years' duration.

His urine was blood-tinged and loaded with mucus.

Microscopic examination revealed an abundance of ammonia, magnesium phosphates, numerous disintegrating pus corpuscles, blood corpuscles and blood shadows.

Repeated examination with the sound gave negative results, but a skiograph taken with a high vacuum hard tube revealed a small calculus which had persistently evaded the sound in previous examinations. Lithotomy was performed and the calculus removed, but the urine failed to return to normal.

Irrigation in turn with Boric Acid, Potassium Permanganate and Silver Nitrate solutions proved unsatisfactory.

Glyco Thymoline irrigations proved satisfactory from the start, and recovery was ultimately perfect.

William L., aged 55. Occupation, saloonkeeper. Had a history of repeated attacks of gonorrhea which were never appropriately treated.

Urine was voided with great difficulty, at frequent intervals and loaded with mucus. Reaction was alkaline, and the microscope revealed an abundance of amorphous phosphates of calcium and magnesium, flat epithelial cells, disintegrating pus corpuscles and indigo crystals.

Examination confirmed diagnosis of chronic cystitis due to urethral stricture and hypertrophied prostate.

Catelectrolysis by the slow method removed the stricture and Bottini's operation relieved the enlarged prostate, but the urine failed to clear up as desired.

The cystoscope showed marked changes in the bladder walls but catheterization of the urether yielded negative results.

Appropriate urinary antiseptics were administered internally and silver nitrate solutions by vesical irrigation with only slight improvement.

Irrigation with twenty per cent. solutions of Glyco Thymoline gave early and continuous improvement until recovery was perfect.

---

A DOCTOR in Texas claims that immediate relief from the desire for whiskey can be obtained by dropping a few drops of the tincture of cinchona on the root of the tongue. The common belief of the profession, a few years ago, was that the tincture of red cinchona was a specific to the whiskey habit.

